# TENS to the Lateral Aspect of the Knees During Stance Attenuates Postural Sway in Young Adults

**DOI:** 10.1100/tsw.2007.279

**Published:** 2007-11-26

**Authors:** Yocheved Laufer, Ruth Dickstein

**Affiliations:** Department of Physical Therapy, Faculty of Social Welfare and Health Studies, University of Haifa, Haifa, Israel

**Keywords:** TENS, posture, stance, stability, child health & human development, neuroscience, sensation and perception

## Abstract

Somatosensory input is known to be essential for postural control. The present study examined the effects on postural sway of sensory input delivered via transcutaneous electrical nerve stimulation (TENS) applied to the knees during stance. Electrodes from a dual-channel portable TENS unit were adhered to the skin overlying the lateral and medial aspect of both knees of 20 young healthy volunteers (mean age 24.0 years, standard deviation 4.0). Postural sway parameters were obtained during static bipedal stance with an AMTI force platform. Four stimulation conditions were tested with eyes open and with eyes closed: no TENS; TENS applied bilaterally; and TENS applied to either the right or the left knee. Participants underwent two eight-trial blocks, with each trial lasting 30 seconds. The order of conditions was randomized for each participant. Stimulation consisted of a biphasic symmetrical stimulus delivered at the sensory detection level, with a pulse duration of 200μsec and a pulse frequency of 100Hz. The application of TENS induced significant reductions in mean sway velocity and in the medio-lateral dispersion of the center of pressure, with no corresponding effect on the anterior-posterior dispersion. These findings suggest that electrical stimulation delivered at the sensory detection level to the lateral aspects of the knees may be effective in improving balance control, and that this effect may be directionally specific.

### INTRODUCTION

Sensory information provided by the visual, somatosensory and vestibular systems are essential for postural control during stance. Ongoing somatosensory input required for postural control is mainly provided by ankle proprioceptors[1,2], cutaneous receptors in the sole of the foot[3,4,5,6,7], as well as receptors in the knee, hip, trunk, and cervical spine joints and muscles[8,10]. Augmentation of somatosensory input during stance is often needed, such as when stance is challenged by external interferences or when the sensory systems that sub serve postural control are affected by pathology[8,9,10,11,12]. For example, individuals with peripheral sensory neuropathy may utilize somatosensory input from their fingertips by light touch in order to compensate for their lower extremity proprioceptive deficits[13].

The application of somatosensory input to enhance postural stability is routinely performed during clinical rehabilitation, primarily through manual contact. The advantage of utilizing these maneuvers is determined by the ability to adjust them in accordance with patient needs. However, manual input is not consistent and the gauging of factors related to its delivery is virtually impossible, given that the amplitude, frequency, and duration of manual contact are not amenable to routine quantification. Furthermore, as manual contact is dependent on direct interaction between the person providing the input and the person in need of somatosensory enhancement, the ability to offer ongoing assistance is limited by practical constraints.

Transcutaneous electrical nerve stimulation (TENS), which involves the pulsatile stimulation of sensory fibers, is a widely applied clinical modality used primarily for the purpose of pain modulation[14,15,16]. Studies have shown that TENS-induced analgesic effects are related to decreases in the activity of noxiously evoked dorsal horn cells stemming from the activation of cutaneous and deep tissue afferent fibers[17,18]. TENS has also been shown to affect upper motor neuron and motor cortex excitability[19,20], which manifests clinically in decreases in spasticity among patients with post-stroke hemiparesis[21,22], and multiple sclerosis[23]. Sensory stimulation also appears to enhance the recovery of functional stability in patients following a stroke[24,25]. Furthermore, the application of TENS to the neck muscles in patients with hemispatial neglect has been shown to improve spatial orientation and postural control[26,27,28,29].

Yet, while TENS is a highly accessible and easily applied modality shown to provide effective somatosensory input modulating central neural activity, its effect on postural stability has rarely been the focus of research attention. In our previous study conducted on this topic, we applied stimulation to the skin overlying the posterior calf musculature, resulting in the attenuation of postural sway as expressed by mean sway velocity[30]. The aim of the current study was to gain further insight into the effects of sensory TENS on stance stability in healthy individuals by applying threshold sensory stimulation along the medio-lateral axis at the knee level. Stimulation at this site was selected on the basis of a previous study in which the application of random electrical noise applied at this location brought about a decrease in postural sway, specifically in the medio-lateral plane[31]. Thus, based on our own as well as on the later cited work, we hypothesized that the administration of TENS would be associated with a decrease in postural sway, with the effect being especially pronounced in the medio-lateral plane. Increasing stability in this plane is of particular interest, as it has been shown to be associated with instability in various pathological conditions[32,33].

### METHODS

Twenty university students (6 men and 14 women), with a mean age of 24.0 years (standard deviation 4.0), volunteered to participate in the study. Participants were blind as to the study objectives. With the exception of one individual, all participants had right leg dominance. Individuals with a history of pathology affecting one of the lower limbs and/or the postural control system (e.g., vestibular disorders) were excluded. The research was approved by the Institutional Ethical Review Committee, and all participants signed an informed consent form in which their rights as subjects were clearly outlined.

#### Instrumentation and Protocol

The study was conducted during the morning hours in a quiet room. Prior to testing, adhesive TENS electrodes (2.5x2 cm) were fastened to the skin overlying the lateral and medial aspect of both knee joints, with the midline of the electrodes positioned parallel to the joint gap line. Except for the specific stimulation sites, the general protocol was similar to the one applied in our previously reported study[30]. Testing was conducted with participants standing in their socks on a portable 50x50x3cm AMTI force plate (Advanced Mechanical Technology Inc., 176 Waltham St, Watertown, MA 02472, USA), while maintaining an 8cm distance between the medial malleoli and a 10° angle between their feet. When assuming the testing position for the first time, the circumference of the feet was marked on the plate in order to assure participants' return to the same foot placement in each trial. The participants' upper extremities were kept folded against their chest throughout all testing trials.

The testing procedure consisted of two blocks of eight trials, with each trial lasting 30 seconds, and a resting period of three minutes between trial blocks. A unique combination of a visual condition and a stimulus condition was used in each trial, and the order of conditions was randomized for each participant. The two visual conditions were: eyes open (EO) and eyes closed (EC). The four stimulation modes were: No TENS (NT); TENS applied bilaterally (BT); TENS applied only to the left knee (TL); and TENS applied only to the right knee (TR).

The stimulation was delivered by a dual-channel battery-operated commercial TENS unit (Elpha 2000 unit, 301 Moodie Drive, Suite 205 Ottawa, Ontario K2H 9C4 Canada). Stimulation consisted of a biphasic symmetrical pulse, with a 200µsec pulse duration and a 100 Hz frequency. Stimulation amplitude was adjusted before the start of each trial in one-milliampere increments and was set at the sensory detection threshold of each participant.

### Data Analysis

Center of pressure (COP) data were collected at a 50 Hz sampling frequency and analyzed offline by a dedicated software program (AMTI Accuswayplus). Postural control was assessed with the following variables: mean COP velocity, which represents the total distance traveled by the COP divided by testing time; amplitude variability of the COP excursion in the anterior-posterior direction; and amplitude variability of the COP excursion in the medio-lateral direction.

For each variable, the mean values of identical trials in the two blocks were used for analysis. Descriptive statistics and repeated measures ANOVA were applied to compare the effects of the four TENS conditions (i.e., NT, BT, TL, TR) and to examine their potential interactions with the effects of vision (i.e., EO, EC). Significance was set at $p<0.05$. Preplanned contrasts were further applied to test the effects of NT (No Tens) against the pooled effect of the other three TENS conditions.

## RESULTS

No significant interactions were observed between the visual conditions and the TENS conditions. Visual condition had a significant effect on all the examined variables, with larger mean sway velocity and sway variability found in both the ML and AP directions for the eyes closed condition than for the eyes open condition. Taken individually, none of the TENS conditions had a significant effect on mean sway velocity. However, the pooled effect of the TENS conditions, as compared to the No TENS condition, resulted in a significant TENS-induced decrease in mean sway velocity ($F(1,18)=7.29$, $p= 0.01$, partial ?2 =0.046). The mean sway velocity in each of the four TENS conditions is depicted in Figure 1.

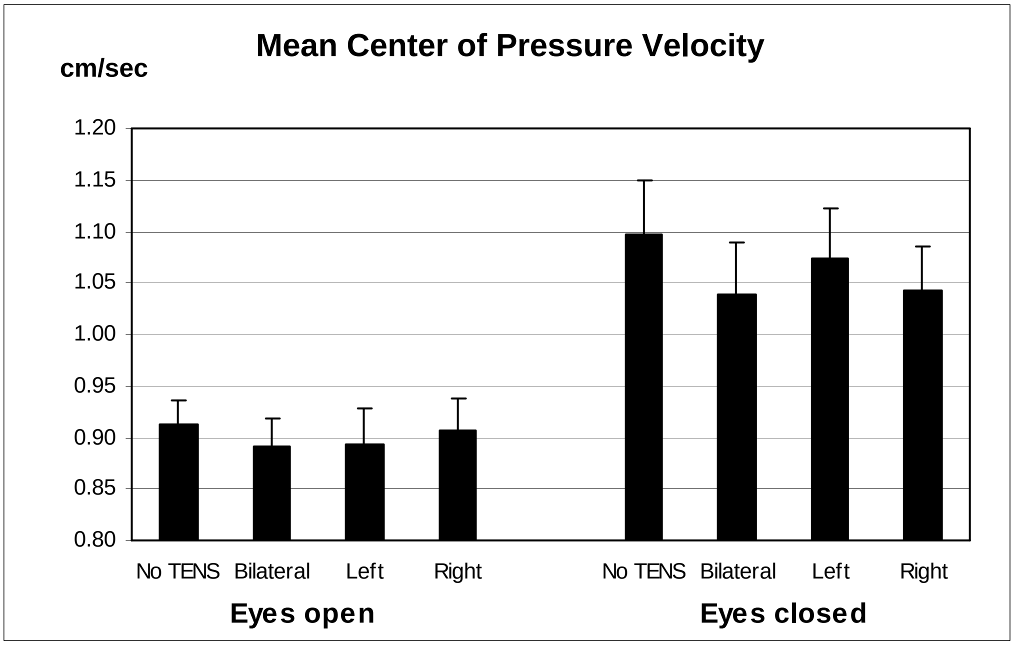

**Figure 1.** Mean and standard deviation of center of pressure sway velocity in each of the four TENS conditions with eyes open and eyes closed.

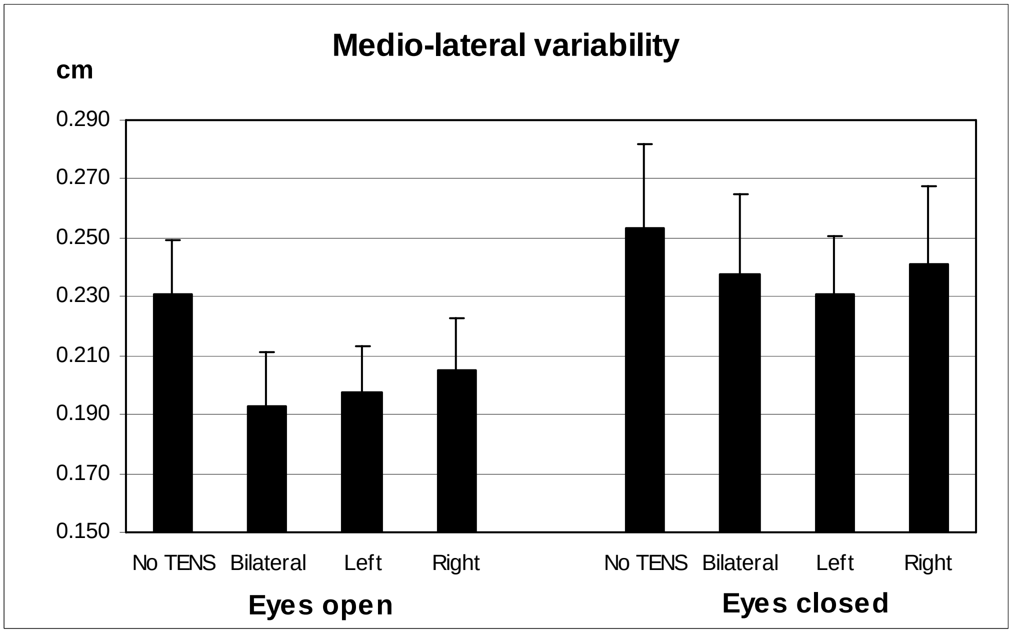

**Figure 2.** Mean and standard deviation of medio-lateral variability in each of the four TENS conditions with eyes open and eyes closed.

A similar trend is noted for the effect of the TENS conditions on medio-lateral variability ($F(3,16)=2.58$, $p=0.089$), with their pooled effect resulting in a significant TENS-induced decrease, as compared to the No TENS condition ($F(1,18)=6.81$, $p=0.018$, partial ?2 =0.04). No similar TENS-induced effect was observed in the anterior-posterior direction. The medio-lateral variability in each of the four TENS conditions is depicted in Figure 2.

### DISCUSSION

The present study demonstrated that COP mean velocity during bipedal static stance is attenuated by TENS applied to the medial and lateral aspects of the knees at sensory threshold intensity. Moreover, the present treatment protocol reduced COP sway variability solely along the frontal plane. In accordance with previous studies, visual input contributed significantly to stability[34,35], with the observed directionally specific effect of TENS found to be independent of the visual condition.

COP movement, expressed as path length (or its equivalent mean velocity), has been determined as the most sensitive postural measure for differentiating between stance conditions or age groups[36]. Yet, changes in this variable alone do not necessarily imply changes in level of stability[37]. More relevant to stability are sway variability measures in the anterior-posterior and/or the medio-lateral directions, which signify the amplitude of the movement around the mean COP location. Decreases in amplitude variability point to a stiffening strategy, which is observed during stance under conditions perceived as threatening to stability, such as when standing on a raised surface[38,39] or while performing a secondary cognitive task demanding attention[40]. It is hypothesized that the prioritization of stability achieved by reducing the amplitude of the sway allows attention to be diverted to whatever secondary condition may be affecting postural control.

The unique finding of the current study lies in the demonstration of a direction-specific effect of the TENS application, as determined by decreases in sway variability in the medio-lateral plane. Human joint movements and related muscular activity are largely expressed along the two orthogonal planes, namely, the anterior-posterior and the medio-lateral planes. It is intuitively assumed during rehabilitation that manually applied somatosensory cues facilitate stability along the plane of sensory input. Thus, for example, when an individual exhibits a tendency to sway/fall sideways, sensory cues are generally provided along the lateral aspects of the body.

In our previous work[30], the application of TENS posteriorly to the skin overlying the medial and lateral gastrocnemius muscles demonstrated a similar reduction in overall sway, but did not induce a similar directionally specific effect. Control of stability in the medio-lateral axis is of particular clinical significance, given that it is affected by a wide range of disorders. For example, elderly fallers, as well as individuals with hemiparesis or Parkinson’s disease, manifest increased medio-lateral instability[32,33]. Yet, the clinical significance of the observed reduction in sway amplitude has yet to be determined.

While these results support the intuitive approach of applying sensory cues in the direction of required stability, they are surprising in terms of the role of the knee musculature during stance. The knee joint, being primarily uni-axial, predominantly controls the height of the body's center of mass, whereas medio-lateral stability is controlled either at the hip with the abductor/adductor musculature or at the ankle with the invertor/evertor musculature. Thus, the process of postural modulation through sensory input must involve central mechanisms, invoking responses in muscles not underlying the area of stimulation.

In several previous studies, sub-threshold random stimulation, either in the form of vibration to the soles of the feet or in the form of electrical impulses to the knees or feet, has been demonstrated to reduce postural sway measures in both non-impaired young and old adults[31,41]. Of particular relevance is the study by Gravelle et al. (2002), who used a form of white noise electrical stimulation to the lateral aspects of the knee during one leg stance and demonstrated decreases in postural sway, as well as in the dispersion of the COP along the medio-lateral plane, similar in magnitude to those found in this study.

It is hypothesized that low-level noise enhances the detection and transmission of weak signals via a mechanism known as stochastic resonance[42]. This concept has been demonstrated in a variety of physical and biological systems, with human subjects demonstrating a lower somatosensory detection threshold as a result of noise application[43,44,45,46]. Thus, it is speculated that the application of noise enhances the use of undetected sub-threshold proprioceptive input that contributes to balance control.

There are two fundamental differences between the TENS used in the present study and electrical noise application, namely, the current stimulus parameters were constant rather than randomized, and intensity was set at the sensory detection level rather than at sub-threshold level. Therefore, it cannot be assumed that stochastic resonance is the underlying mechanism of the observed effect. It is more likely that the TENS enhances somatosensory input through its direct effect on the afferent nerve fibers. Experimental evidence indicates that the CNS is informed on the position and movements of the knee via ensemble coding mechanisms, rather than via modality specific pathways[47]. These coding mechanisms involve inputs from the skin, ligaments, capsular and muscle receptors, acting as a final common path that conveys information to the CNS[48]. Thus, although the effect of TENS is primarily exerted via cutaneous afferents, the potential influence on proprioceptive information to the CNS is highly plausible. As TENS inputs are mediated via central, mainly cortical, networks, which affect motor cortex excitability[20,49], higher-order effects may also contribute to the TENS-induced changes in postural sway. It must be, however, noticed that the small effect sizes (partial $\eta^2$ were below 0.10) are below what might be considered clinically useful. Yet, this preliminary study examined the effect of a very short stimulation period in young and healthy adults with intact postural stability. These factors most likely minimized the stimulation effects.

### CONCLUSIONS

The results support our previous study and point to the potential of TENS applied at very low stimulation doses to affect postural sway, indicating that the location of the stimulus may have a directionally specific effect. Due to the limited nature of this study, however, wide-ranging interpretations should be avoided. Future studies should explore questions related to the clinical significance of these findings, particularly in populations with postural control impairments. The low cost and ease of application of TENS render its use extremely accessible. Additional research should also be conducted on various aspects of treatment protocol, such as stimulation parameters (e.g., application site and time, pulse width, frequency, and intensity), which may further enhance the effectiveness of treatment.
